# Reticular activating system of a central pattern generator: premovement electrical potentials

**DOI:** 10.1002/phy2.129

**Published:** 2013-10-24

**Authors:** Jesus A Tapia, Argelia Trejo, Pablo Linares, J Manuel Alva, Rumyana Kristeva, Elias Manjarrez

**Affiliations:** 1 Institute of Physiology, Benemérita Universidad Autónoma de Puebla14 Sur 6301, Col. San Manuel, Puebla, Puebla, CP 72570, México; 2 School of Biology, Benemérita Universidad Autónoma de Puebla14 Sur 6301, Col. San Manuel, Puebla, Puebla, CP 72570, México; 3 Neurological Clinic, University of FreiburgBreisacherstraße 64, 79106, Freiburg, Germany

**Keywords:** Dorsal horn neurons, locomotion, scratch reflex, scratching, slow oscillations, spinal rehabilitation

## Abstract

For the first time, here we characterize a bulbar reticular activating system (RAS) of neurons in decerebrate, deafferented and decerebellated cats producing a premovement electrical potential that we named obex slow potential (OSP). The OSP occurs about 0.8 ± 0.4 sec prior to the onset of a fictive-scratching-episode. Here, we describe two classes of bulbar neurons, off-on, which are silent but exhibit a 80 ± 56 Hz firing discharge at the beginning of (and during) the OSP, and on-off interneurons, with a 27 ± 14 Hz firing activity that stops at the beginning of (and during) the OSP. We suggest that these OSP-associated neurons belong to a descending RAS, which contributes to the activation of the spinal central pattern generators.

## Introduction

A fundamental problem in neuroscience is the understanding of neuronal mechanisms by which the movement is originated. These mechanisms have been mostly investigated in forebrain structures by means of premovement electrical potentials recorded in the brain. In 1965, Kornhuber and Deecke published a seminal study about an electroencephalographic (EEG) potential of the human brain, called the readiness potential or bereitschaftspotential (BP), which occurs ∼1 sec before the onset of a self-initiated movement (see [Kristeva et al. 1979] and [Kristeva and Kornhuber 1980]). Following this remarkable discovery, in 1974, DeLong and Strick reported the occurrence of electrical activity of medium spiny neurons from the basal ganglia in monkeys prior to the movement onset. In 1979, Brinkman and Porter also detected firing activity of neurons from the supplementary motor area (SMA) in monkeys before the movement. In the same context, Romo and Schultz 1987 verified that both classes of neurons from the SMA and the basal ganglia exhibit firing activity up to 3 sec prior to the onset of self-initiated movements in the monkey. Moreover, after the original finding of Kornhuber and Deecke in 1965, other seminal study of EEG performed in humans suggested that the unconscious initiation of a free voluntary act occurs between the beginning of the readiness potential and the movement onset (Libet et al. 1983). This last study was recently extended by Fried et al. (2011), who recorded the unitary activity of neurons from the SMA while human subjects performed self-initiated finger movements.

Premovement modulation of other neurons has been shown during the scratching “latent” period in the cat (i.e., the period between stimulation and rhythmic motor activity). For example, L4/L5 spinal interneurons (Berkinblit et al. 1978a,b) and ventral spinocerebellar tract neurons modulate firing long before the onset of rhythmic activity (Arshavsky et al. 1978a). Arshavsky et al. (1978b) reported a small population of lateral reticular nucleus neurons (spinal-reticular cerebellar pathway) that are inhibited in the latent phase of scratch. Rubrospinal tract neurons become tonically active during the latent period (Arshavsky et al. 1978c). Some fastigial nuclei (cerebellar) neurons were inhibited during the latent period (Antziferova et al. 1980). Moreover, interpositus nucleus neurons exhibit tonic activation during the latent period (Arshavsky et al. 1980).

The above-mentioned studies show that premovement electrical potentials have been extensively characterized for regions involving the forebrain. However, little is known about premovement electrical potentials associated with the activation of the spinal central pattern generators (CPG) and the brainstem.

The purpose of this study was to characterize unitary electrical activity of neurons as well as the brainstem neuroanatomical regions related to premovement electrical potentials prior to the onset of spinal CPG activation during scratching in cats.

## Material and Methods

We performed experiments in eight decerebrate and paralyzed cats. We recorded surface field potentials as well as the neuronal unitary activity of the obex and the rhomboid fossa. Surface potentials were obtained by means of an array of 32 electrodes. Neuronal unitary activity was recorded by means of single microelectrodes.

### Surgical preparation

Experiments were carried out in accordance with the European Communities Council Directive of 24 November 1986 (86/609/EEC), the guidelines contained in the National Institutes of Health Guide for the Care and Use of Laboratory Animals (85–23, revised in f1985) and the “Norma Oficial Mexicana NOM-062-ZOO-1999.” Moreover, the Institute of Physiology from the Benemerita Universidad Autonoma de Puebla, Mexico, approved this study. Experiments were performed in eight adult cats (2.0–3.5 kg). The animals were gently handled and introduced in a comfortable anesthesia-induction-box at a temperature of 22–29°C and 40–70% relative humidity. Anesthesia was induced with a mixture of isoflurane (Sofloran, PiSA farmaceutica, Puebla, Mexico) and oxygen. Atropine (0.05 mg/kg) and dexamethasone (2 mg/kg) were applied at the beginning of the experiment. The level of anesthesia was verified throughout the surgery by measuring the arterial blood pressure and the lack of withdrawal reflexes. Later, arterial blood pressure was monitored by a cannula inserted in the right carotid artery. We also cannulated the radial vein in order to administer a buffer solution during the entire experiment (NaHCO3 100 mmol/L and glucose 5%, at a rate of 5 mL/h). Dextran and saline solutions were administered when necessary to sustain a physiological blood pressure (80–120 mmHg). A bilateral pneumothorax was done after the induction of paralysis and mechanical ventilation.

Medial gastrocnemius (MG), lateral gastrocnemius plus soleus (LGS), and tibialis anterior (TA) were dissected in both hind limbs. A laminectomy was performed at cervical C1–C3 and lumbosacral L4-S1 spinal segments; we removed the dura mater of the exposed spinal segments. After this, the animal was transported and mounted in a stereotaxic frame (Narishige, Tokyo, Japan). We performed a mechanical decerebration at a precollicular-postmammillary level. After the decerebration we discontinued the anesthesia, paralyzed the animal using pancuronium bromide (Pavulon; Organon, Roseland, NJ) and artificially ventilated. We performed a decerebellation by transecting the inferior and superior cerebellar peduncles and exposed the rhomboid fossa and the obex. Animals were euthanized at the end of the experiment with an overdose of pentobarbital (175 mg/kg, i.v.). Zero blood pressure indicated that the animal was euthanized.

### Electrophysiological recordings

The scratching reflex was elicited by mechanical stimulation of the ipsilateral pinna, neck or head. For the mechanical stimulation, we employed a Chubbuck mechanical stimulator-transducer (Chubbuck 1966) and a Master-8 pulse generator, A.M.P.I., Jerusalem, Israel. Short-lasting (0.2 sec) mechanical stimulation was applied to elicit the scratching episodes. Previously, we applied a cotton ball soaked with d-tubocurarine (0.1%) (see the incidental observation of Feldberg and Fleischhauer in 1960) on the surface of the C1–C2 spinal segments to easily produce scratching episodes with short-lasting mechanical stimulation of the pinna.

We recorded the electroneurographic activity of flexor and extensor nerves, and surface potentials from the obex on a Synamps2 EEG amplifier (NeuroScan, Compumedics, Charlotte, NC) using a multielectrode array of 32 monopolar Ag-AgCl electrodes (200-μm diameter). Similarly, we recorded the cord dorsum potential (CDP) of the L6 spinal segment with a single monopolar electrode.

In all the experiments, we used glass microelectrodes (7–15 MΩ) filled with NaCl (1.2 mol/L) to record extracellular unitary activity of reticular neurons before and during the fictive scratching episodes in the vicinity of regions which exhibited the maximal amplitude of the OSP.

#### Histology

Microelectrode placements were verified by histology. At the end of each corresponding experiment, the micropipettes were left in the recording site. The animal was euthanized with an overdose of pentobarbital (175 mg/kg i.v.) and perfused with 10% formalin. After the complete fixation and dehydration, the bulbar tissue was placed in a solution of methyl salicylate for clearing. Then, we segmented and subsequently photographed the bulbar sections containing the microelectrode. A trajectory was obtained for each microelectrode and the sites of the recordings were superimposed onto these trajectories.

### Data analysis

#### Characterization of the obex slow potential

To measure the time interval between the obex slow potential (OSP) and the onset of flexor electroneurographic activity (i.e., the initialization of the scratching episode), we averaged 156 scratching episodes obtained in eight cats. We obtained windows of 2000 msec prior to the onset of flexor activity and 1000 msec of post onset activity. The main reason for this asymmetry was the high variation in the flexor tonic phase duration, ranging from 1 to 7 sec. Before the recordings, we carefully placed the multielectrode array in the same position in all the experiments taking into account the obex as an anatomical reference. We analyzed the averaged and standard deviation of the OSP recorded with electrodes 11 or 19, because both electrodes held the closest position to the obex. With this data for each electrode, we performed a spline 3D reconstruction of the spatial distribution of the OSP maximal amplitude. The nonparametric Spearman's rank coefficient method was used to test for significant correlations between the brainstem OSP and the spinal CDP (*P* < 0.01).

#### Change point of neuronal onset and offset

Off-line data analysis was performed using a sliding window procedure, similar to the procedure used by Apicella et al. (1992). In this analysis, we obtained the change point of neural activity for each single neuron, using the nonparametric one-tailed Wilcoxon signed-rank test. In these tests, we successively compared two trials treated as matching samples. One was a Control Window (CW) obtained from the basal activity of the neuron (2–2.5 sec). The second window (Onset Window, OW) consisted of a time interval of 250 msec that was swept in steps of 25 msec throughout the time period of a suspected change. The beginning of the OW epochs was proposed qualitatively based on the appearance of the peri-scratching histogram. The last OW epoch was the interval which exhibited the highest spike count of the entire scratching episode (∼200 msec after the onset of the flexor activity). A Wilcoxon test was computed at each step of 25 msec. The time at which the onset of neural activity occurred was mid-window of the first of five consecutive steps, showing a statistical increase between the control and the OWs (*P* < 0.01). On the other hand, the offset of activation was obtained in a similar manner by searching the significant decrease in neuronal discharges (*P* < 0.01).

## Results

### Distribution of the OSP

**Figure 1:**
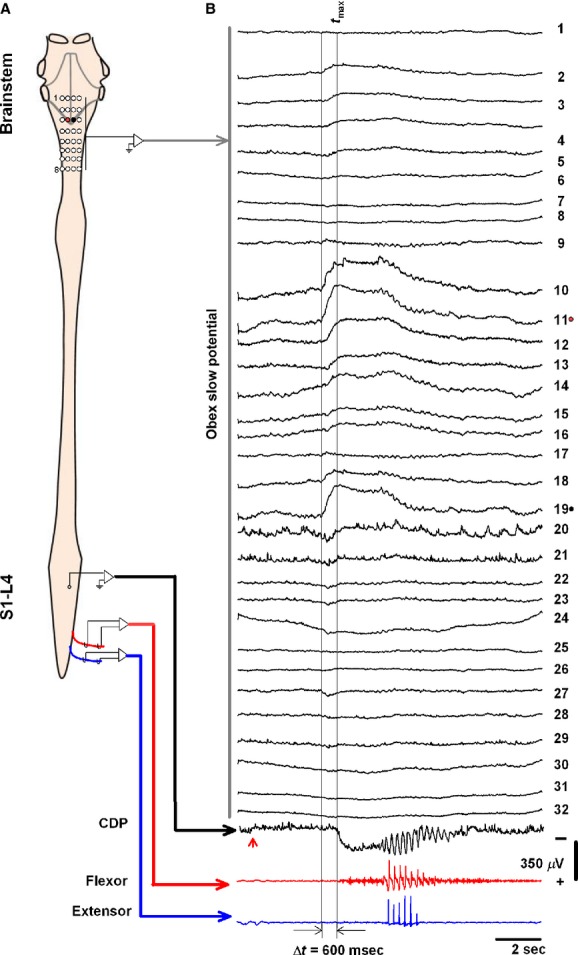
Obex slow potential (OSP) recordings before, during, and after a fictive scratching episode. (A) Scheme of the experimental arrangement, the red circle in the multielectrode scheme represents electrode number 11, while the black circle is the electrode number 19. (B) DC recordings of the OSP during a scratching episode (1–32 monopolar surface recordings). The vertical lines indicate the beginning of the OSP and the onset of the flexor tonic discharge. Note a 600-msec delay between the OSP and the cord dorsum potential (CDP) recorded on the lumbar spinal cord. The lower red arrow head marks the beginning of the mechanical stimulation (lasting 0.2 sec) to the pinna.

Scratching episodes were elicited by means of a short mechanical stimulation (see Method and Materials) applied on the pinna. Figure 1 shows Direct Current (DC) recordings of the obex and C1–C2 CDPs (electrodes 1–32), L6 segment dorsal potential (electrode 33) and electroneurograms (electrodes 34 and 35) during a single fictive scratching episode. This figure shows recordings of slow potentials developed at the vicinity of the obex indicated by the numbers 10, 11, and 12. Hence, we termed this activity as “OSP”. Because the OSP occurred before the scratching episode (red and blue traces in Fig. 1), and even before the associated DC shift of the spinal CDP, we considered the OSP as a premovement potential. A grand average obtained from eight experiments shows that the OSP occurs 0.8 ± 0.4 sec prior to the onset of fictive scratching. An inspection of Figure 1 indicates that the distribution of the OSP is clearly restricted to the lower parts of the medulla and the upper segments of the spinal cord (C1–C2). This OSP starts before scratching but it is maintained during the whole scratching episode, and it subsides shortly after the end of the scratching episode.

**Figure 2:**
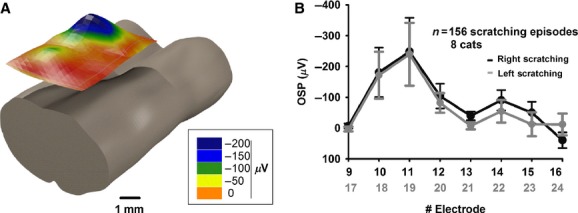
Distribution of the obex slow potential. (A) The upper colored figure is a 3D representation of the voltage recorded at each electrode at tmax (See details in text). The lower figure is a 3D reconstruction of an upper spinal segment and the medulla. (B) Longitudinal distribution for the mean amplitude of the obex slow potential (OSP) recordings, the black line is for left scratching, and the gray line is for right scratching.

The spatial distribution of maximum amplitude for the OSP is represented in a 3D reconstruction (Fig. 2A). We observed that the maximal amplitude of the OSP occurred in the vicinity of the obex and rostrolateral regions (1 mm rostral to the obex and 1 mm lateral to the obex). This is also illustrated in Figure 2B, in which there is a peak of maximum voltage of −248 ± 110 μV for the OSP associated with left scratching. While the maximum voltage recorded during right scratching was −239 ± 102 μV, there are no significant differences between left and right voltage amplitude distribution for the OSP.

### The OSP amplitude is highly correlated with the lumbar-CDP amplitude

**Figure 3:**
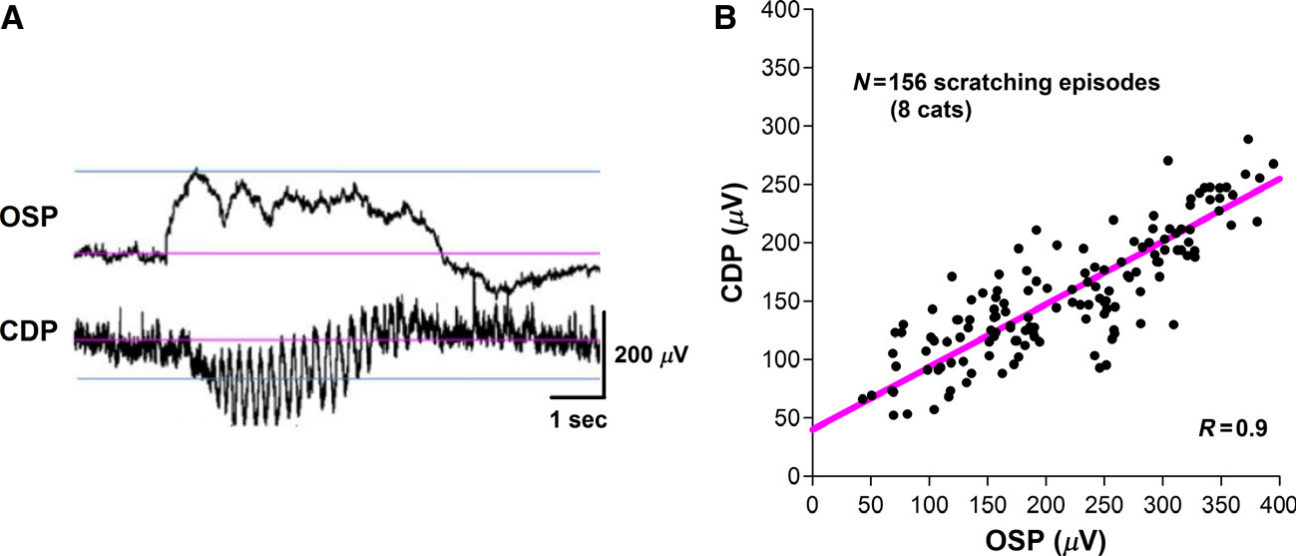
(A) We measured the peak amplitude of the obex slow potential (OSP), while we measured the largest DC peak of the cord dorsum potential (CDP) during the tonic phase of the fictive scratching. (B) Correlation between the OSP and the L6 CDP peak amplitudes.

The OSP exhibits a negative component, which rises before the onset of the scratching cycle; this OSP also exhibits a slow negative potential once the tonic phase has already started. Figure 3A illustrates simultaneous recordings of the OSP and the DC shift of the lumbar L6 CDP. Note that the onset of the OSP occurred before the onset of the CDP. The horizontal lines in Figure 3A indicate the maximal amplitude of the OSP (upper line) and the mean amplitude of the CDP (lower line); Figure 3B shows a scatter plot with these measures obtained from 156 scratching episodes of eight experiments. Each point represents a single scratching episode. We obtained a Spearman's rank correlation coefficient *R* = 0.9, *P* < 0.01.

### Single-unit extracellular recordings

**Figure 4:**
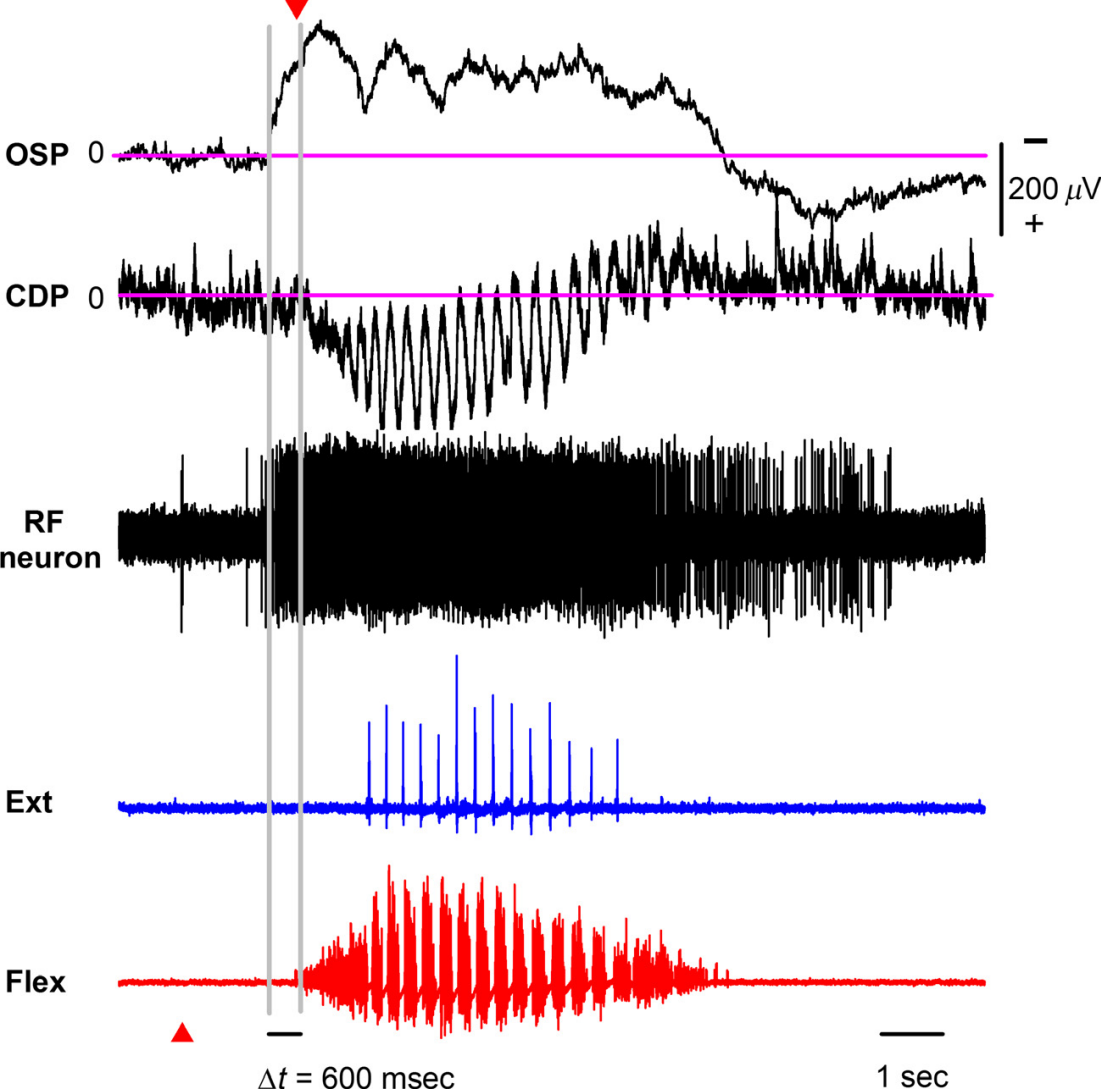
Extracellular recording of a reticular formation (RF) neuron during a fictive scratching episode. The upper trace is the surface recording of the obex slow potential obtained in the vicinity of the obex. The next trace depicts an electrical recording of the spinal cord surface at L6 level (i.e., the Cord Dorsum Potential, CDP). The lower black trace is the extracellular unitary recording of the RF neuron. Electroneurograms are indicated with a blue trace for the extensor (Ext) and a red trace for the flexor (Flex) activity. The recorded neurons start firing tonically 600 msec prior to the onset of the scratching episode. We termed this firing profile as off-on.

Extracellular unitary recordings were made in the region in which the OSP of maximal amplitude was detected. We recorded extracellular unitary activity of neurons before and during the onset of fictive scratching. We found three types of neuronal responses. The first type of neurons exhibited a background discharge with a very low rate (∼1 Hz) before the onset of the scratching episodes. Yet, after the stimulation and before the onset of the tonic flexor activity, these neurons increased their firing rate from 1 Hz to a mean of 80 ± 56 Hz (Fig. 4). This increased discharge pattern persisted during the whole scratching episode and subsided when scratching ended. Neurons which exhibited this response during scratching were termed “off-on” neurons (Fig. 4).

**Figure 5:**
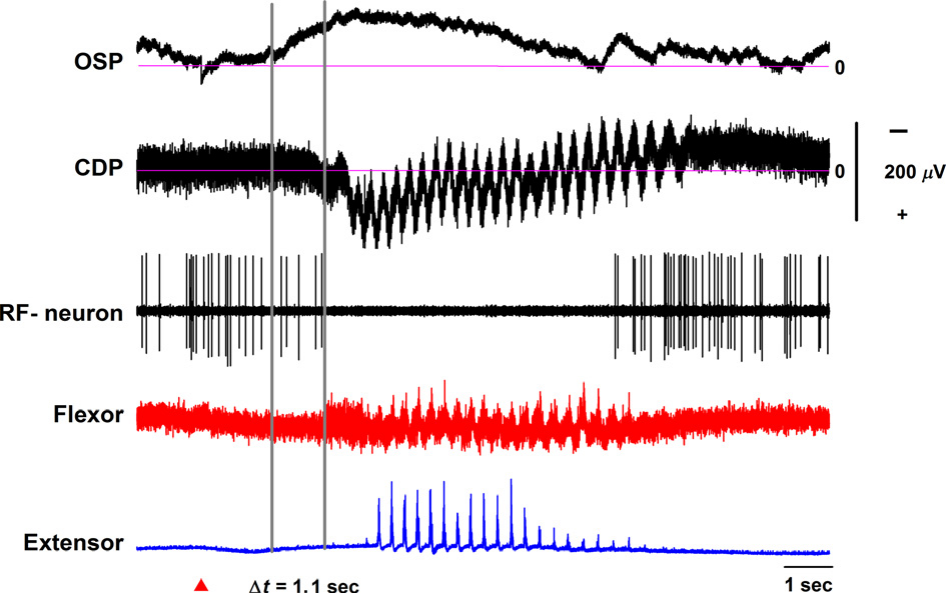
Extracellular recording of a reticular formation (RF) neuron during a fictive scratching episode. From top to bottom, the traces represent the obex slow potential (OSP), the L6 cord dorsum potential (CDP), the extracellular recording of a single RF neuron, and the flexor and extensor electroneurograms. The activity of this neuron showed a tonic firing, yet, it subsides 80 msec prior to the onset of the scratching reflex. Hence, we termed this firing profile as on-off.

We observed another discharge pattern: neurons which exhibited regular background firing with a mean of 27 ± 14 Hz frequency rate. These neurons ceased their firing activity at the beginning of the scratching episode and returned to their background firing activity when the scratching episode ended. For the neurons of this class, the “silent” period lasted the whole scratching episode. We termed this class of neurons as “on-off” neurons (Fig. 5).

We found an additional type of neurons; these neurons showed an unmodulated tonic firing before, during, and after the scratching episodes, hence, these neurons were classified as “tonic neurons”.

### Neuronal discharge frequency

**Figure 6:**
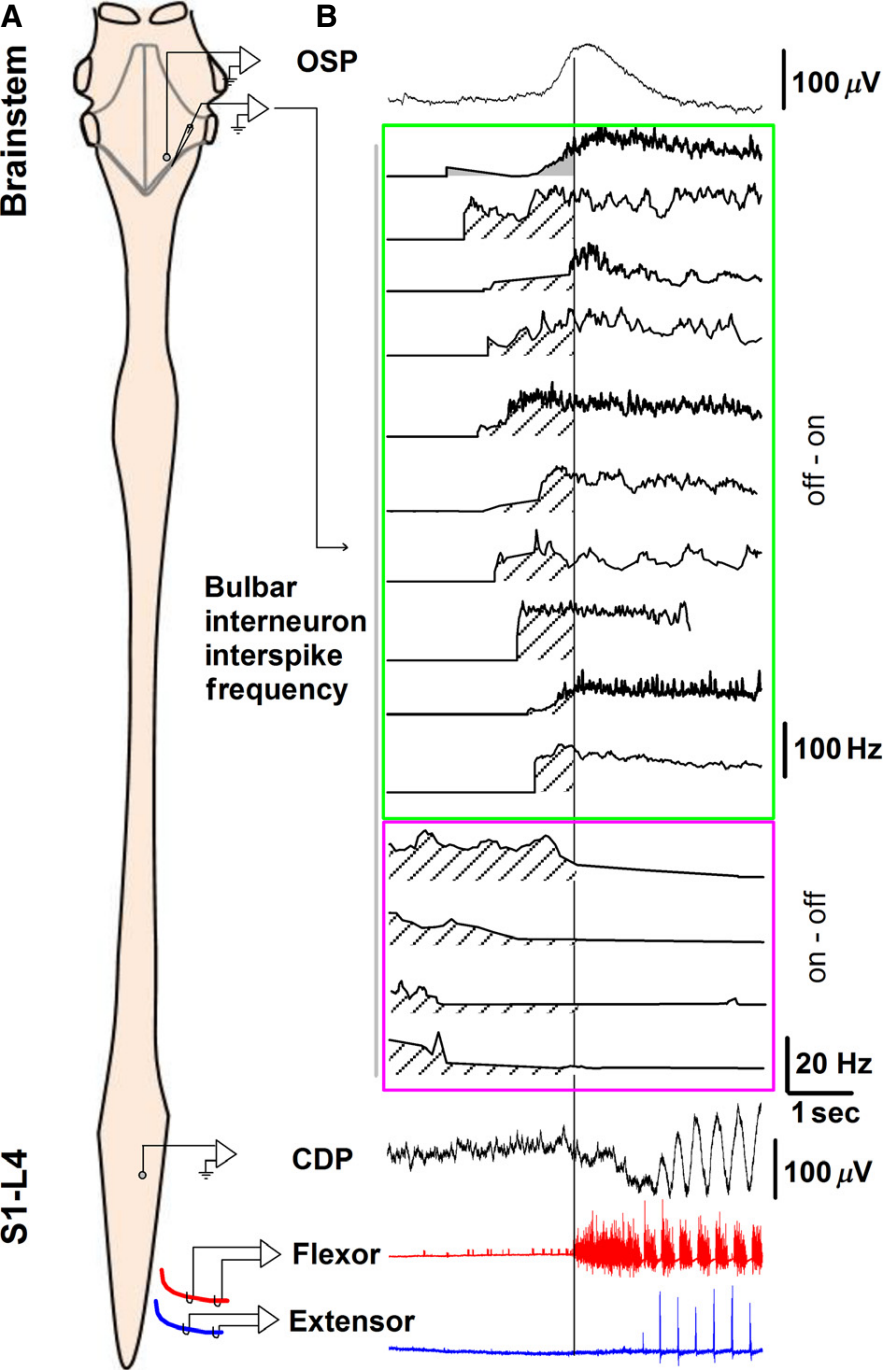
Pooled data of the frequency profile of reticular formation interneurons before the onset of the scratching activity. (A) Schematic diagram of the surface electrodes position, and the site for the extracellular recordings. (B) Frequency profile. The uppermost recording is the obex slow potential (OSP). The green box encloses neuronal frequency profiles of neurons belonging to the off-on activity pattern. The magenta boxed recordings correspond to the on-off neurons. The next trace depicts the L6 cord dorsum potential (CDP). The red and blue traces represent the flexor and extensor electroneurograms.

We obtained the instantaneous discharge frequency of 10 off-on and 4 on-off neurons (Fig. 6). This analysis shows a sudden increase in discharge frequency of the off-on neurons prior to the onset (vertical line in Fig. 6) of the flexor tonic activity. However, in the case of the on-off neurons, the discharge frequency decreases before the scratching onset (vertical line in Fig. 6). Further differences were observed in the mean discharge frequency of both types of neurons. Off-on neurons exhibited a 1 ± 0.7 Hz pre scratching background frequency and a 80 ± 56 Hz frequency during scratching; while the on-off neurons had a 28 ± 15 Hz pre scratching background frequency and a 0.1 ± 0.08 Hz frequency during scratching. Tonic nonmodulated neurons exhibited a 122 ± 25 Hz frequency during the whole recordings and were unperturbed during scratching (see summary in Fig. 9).

### Results obtained from the analysis of “change point of the neuronal activity”

**Figure 7:**
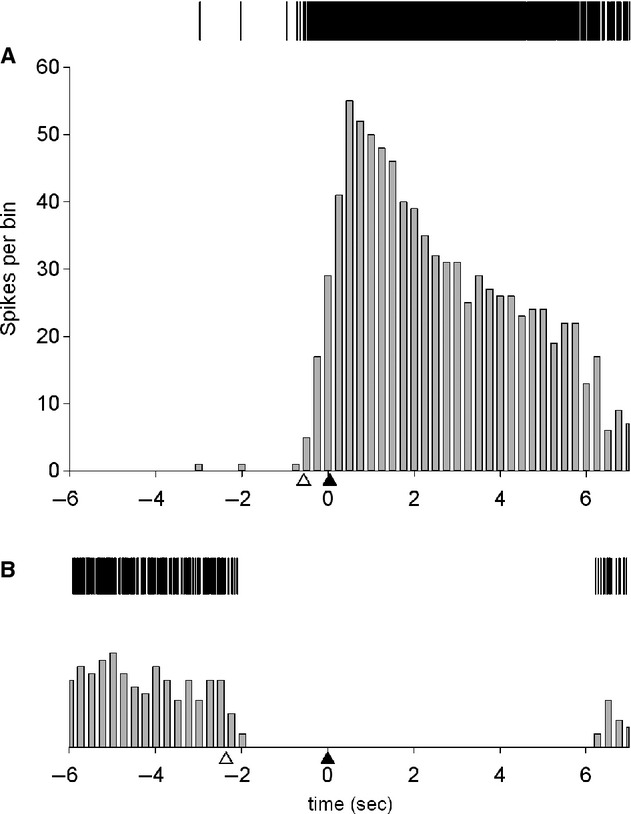
Change point calculated for neurons belonging to both groups (off-on and on-off). (A) Raster and spike count histogram for an off-on neuron. Note the sudden increase in spike count prior to the onset of scratching. (B) Raster and histogram of spike count for an on-off neuron. Change points are marked with a white triangle, while the black triangle represents the onset of flexor activity. Details of the methods can be found in the text.

**Figure 8:**
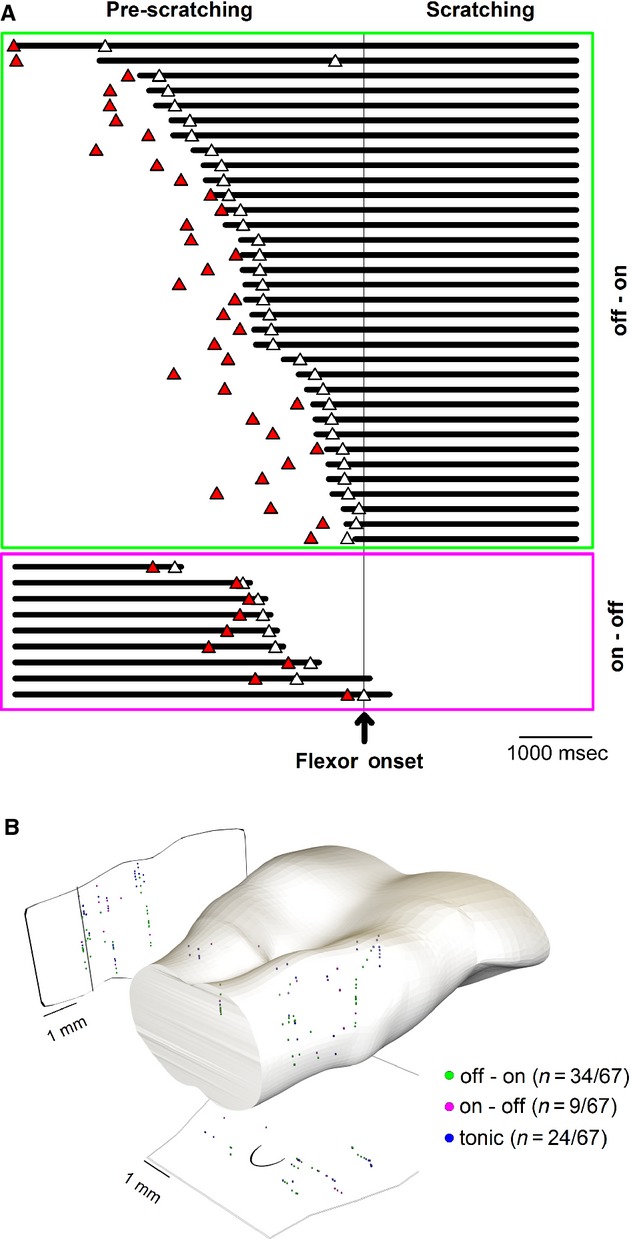
Discharge distribution of 43/67 neurons and its spatial localization within the medulla at the level of the obex. The bursting activity of each neuron is represented by a solid line. (A) Raster plots of all the recorded interneurons associated with the obex slow potential (OSP). The neurons belonging to the off-on class increased its firing activity prior to the onset of flexor activity (Green box). On the other hand, almost all the neurons belonging to the on-off class decreased their firing activity prior to the onset of the flexor activity (Magenta Box). Red triangles represent the time at which the mechanical stimuli (0.2-sec duration) was applied. In two cases, we recorded a few discharges after the beginning of the flexor tonic phase, yet this activity was below 3 Hz. (B) 3D histological reconstruction of the localization of the recorded neurons. The reticular formation was the area in which the majority of the neurons were located. The vertical line on the leftmost medulla projection represents the obex position.

Statistical analysis was performed to calculate the time at which the neurons could be considered as “activated” or show a statistical difference against their background activity. This method is called “change point of neuronal activity” and is illustrated in Figure 7 (see Material and Methods section for details). We applied this method to quantify the significant timing in which the off-on neurons started to fire relative to the onset of the scratching episode. In Figure 8A (upper panel), the change points computed for the all the recorded off-on neurons are indicated with white triangles (mean timing −1391 ± 1008 msec). The importance of these “change points” is that they provide a statistical significant measurement for the timing of the beginning of the neuronal firing activity. In Figure 8A (lower panel), the change points computed for the all the recorded on-off neurons are also indicated with white triangles (mean timing −1417 ± 734 msec). The importance of these “change points” is that they provide a statistical significant measurement for the timing of the ending of the respective neuronal firing activity.

### Intramedullary localization of off-on and on-off neurons

We identified the distribution of the recorded neurons by means of the histology of the medulla obtained from every experiment. We found that both the off-on and the on-off neurons were located at the reticular formation (RF), at the level of the obex (P12–12.5), and at a depth of 2222–4118 μm. The spatial distribution of both types of neurons clearly shows a superimposed localization; thus suggesting that they are not segregated in two nuclei (Fig. 8B).

### Summary of frequency rates for the off-on, on-off and tonic neurons

**Figure 9:**
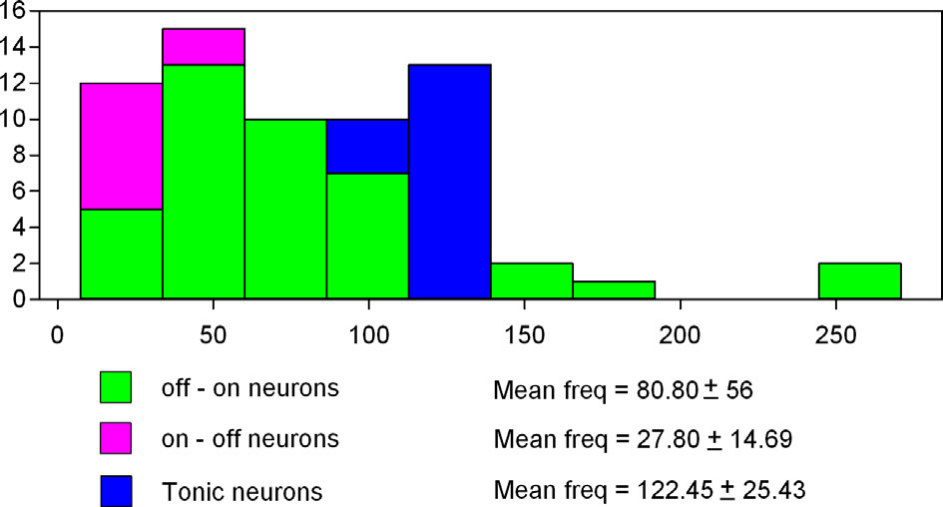
Mean spike frequency (Mean freq, in Hz) histogram for the off-on, the on-off, and the tonic obex slow potential (OSP)-related neurons during scratching.

We pooled in a histogram (Fig. 9) the frequency rates of the off-on, on-off, and tonic neurons during scratching for all eight cats (67 neurons). We found that the off-on neurons fired at a mean frequency of 80 ± 56 Hz, the on-off neurons fired at 27 ± 14 Hz, and the tonic neurons at 122 ± 25 Hz.

## Discussion

In this article, we described the occurrence of a slow pre-movement potential, which appears 0.8 ± 0.4 sec before the onset of the fictive scratching and persists the whole episode duration. This potential was recorded in the vicinity of the obex and the caudal part of the rhomboid fossa and was termed OSP. Furthermore, we recorded the extracellular unitary activity of single neurons in the region where the OSP exhibited its maximal amplitude. These neurons exhibited a sudden increase or decrease in their background firing rate before the onset of the scratching episode; thus suggesting that they are participating in the activation of the spinal CPG for scratching. In this context, it is possible that both classes of neurons could belong to the reticulospinal system.

Deliagina et al. 2000, studied the activation of a possible command neuron circuit in the pontine RF of the lamprey during swimming. These neurons showed a tonic firing during the entire swimming episode. Other studies demonstrated the existence of neurons within the subthalamic and mesencephalic motor region. Electrical stimulation of these structures elicited locomotion in the decerebrate (Shik et al. 1966), and intact cat (Mori et al. 1989). Stimulation at this region also produces swimming in the lamprey (Sirota et al. 2000). However, there is evidence of other groups of neurons that could contribute to the initiation of movement but not to its ongoing activation.

In our experiments, we found three classes of brainstem neurons, two of which were clearly related to the activation of the scratching CPG. One of these types of neurons increased its frequency discharge about 1391 ± 1008 msec prior to the scratching onset and which was maintained throughout the complete episode. This behavior is consistent with the command neurons predicted by Sherrington (1906). In this context, we propose that our recorded brainstem neurons could share characteristics of the command neurons, which receive a brief tactile input and translate it to a tonic discharge, which in turn descends via the reticulospinal pathways in order to activate the CPGs. Experimentally, other command neurons have been found for several species, for example in the lamprey (Deliagina et al. 2000), tadpole (Buhl et al. 2012), felines (Jankowska et al. 2011), and primates ([Romo and Schultz 1987]; [Baker 2011]). In their experiments, Romo and Schultz (1986) show unitary activity of neurons, which also appear before the onset of voluntary movements. These neurons could be involved in the commanding and the initiation of movement while also producing the premovement potential recorded by Kornhuber and Deecke (1965). However, despite that plausible functional correlation, the authors void the statement that SMA neurons are commanding the onset of movements, as other structures also exhibit activity prior the beginning of movements (Schultz 1986). As we stated earlier, our preparation does not receive cortical nor mesencephalic projections because the animal was decerebrate. This allows us to suggest that reticulospinal neurons are command neurons for the motor activity of scratching.

We found three discharge patterns in the neurons recorded. Furthermore, those groups of neurons are mixed spatially (Fig. 8B), this stresses the great diversity and heterogeneity of the RF. This is due to the several functions executed within the RF such as respiration (Cohen 1979), cardiovascular control (Lima et al. 2002), and nociceptive integration (Gauriau 2002). This diversity explains the difficulty to activate the locomotion or scratching CPGs by means of electrical stimulation. RF microstimulation would activate several neural groups, which exert excitatory and inhibitory inputs to the CPG. We can imagine the off-on neurons as carrying excitatory inputs, while the on-off neurons would project inhibitory synapses. The stimulation of both groups would exert a mixed signal to the CPG, thus impeding its correct activation.

The reticular system is a group of neurons distributed in the brainstem, the mesencephalon, and the diencephalon. In 1949, Moruzzi and Magoun proposed that neurons from this group belong to the reticular activating system (RAS), which is responsible for the awaking state in both cats and humans. Presumably this ascending RAS can elicit responses in thalamic and subsequently in cortical neurons, thus generating a generalized activation of cortical neuronal populations in the whole brain and the concomitant awaking state. In this context, it was suggested that transition from sleep to arousal is mediated by this ascending RAS. Because the arousal state is related to muscular activation, that is, an enhancement of the muscular tone, it was also suggested that an additional descending RAS could be responsible for the activation of spinal motoneurons.

The idea that a descending RAS produces responses in spinal neurons opens the hypothesis that there is a descending RAS responsible for the activation of spinal CPG. The results presented here support this hypothesis.

The study of the RAS is an intriguing area of research, initiated by Moruzzi and Magoun (1949). These authors produced comas in cats by using coagulation to destroy the brainstem RF. In other experiments, they found that stimulation of this region evokes desynchronization of the EEG, simulating the arousal reaction to sensory stimuli, thus awaking the animals from normal sleep. However, this hypothesis has been controversial after some experiments of selective lesions of neurons in the RF. Garcia-Rill et al. 2013, proposed an interesting functional role for the RF. These authors suggested that the continuous sensory input will modulate coupling and could induce gamma band activity in the RAS that may participate in the processes of preconscious awareness, and provide the essential stream of information for the formulation of many of our actions. In this context, this study (see the frequency band of the off-on neurons, Fig. 9) is consistent with evidence that the RAS exhibits gamma activity, which has been considered as a mechanism to stabilize coherence related to arousal (for review see [Urbano et al. 2012]). In line with these studies, it is tempting to speculate that “gamma activity” mediated by the off-on neurons could provide a mechanism to elicit the CPG activation. Moreover, it is tempting to speculate that gamma activity of the off-on neurons recorded at the obex could stabilize coherence in the CPG circuits.

Also, the RF has been related to the awake-sleep cycles (Torterolo and Vanini 2010). This function was termed ascending activating system and is highly correlated with a neural “tonus” needed for the execution of wakeful tasks (Klingberg et al. 1989). We suggest that the RAS plays an important role in the activation of the CPGs via the obex off-on and on-off neurons, which select the neuronal circuits to be activated. This possibility is consistent with previous studies about microstimulation of the medullary RF in intact, unanesthetized cats (Drew and Rossignol 1990a,b), in which complex movements of more than one limb and the head were evoked. Most likely happening during the massive stimulation of the RF, both on-off and off-on neurons are activated, giving place to complex movements. It is tempting to speculate that a selective stimulation of the off-on neurons could produce activation of the CPG.

The on-off neurons described in this study could be involved in the selection of locomotion or scratching. This could be possible because Sherrington originally noted that the locomotion and scratching are mutually exclusive with stepping movements always ceasing when scratch was evoked, suggesting that with activation of scratching, the activity of the reticular spinal system influencing locomotion is suppressed (see review in [Whelan 1996] and Discussion in [Orlovsky 1970; Pavlova 1977]). This is consistent with the fact that reticulospinal neurons can be modulated during the activation of the CPG spinal circuits (Orlovsky 1970; Pavlova 1977).

In conclusion, we identified for the first time a bulbar RAS of neurons firing at 80 ± 56 Hz and 27 ± 14 Hz, which are activated before the onset of the fictive motor task of scratching in the cat. These neurons are also correlated to an OSP that occurs prior to the onset of the fictive movement.
